# Grapevine Leafroll-Associated Virus 3 Genotype Influences Foliar Symptom Development in New Zealand Vineyards

**DOI:** 10.3390/v14071348

**Published:** 2022-06-21

**Authors:** Kar Mun Chooi, Vaughn A. Bell, Arnaud G. Blouin, Daniel Cohen, Dion Mundy, Warwick Henshall, Robin M. MacDiarmid

**Affiliations:** 1 The New Zealand Institute for Plant and Food Research Limited (PFR), Private Bag 92169, Auckland 1142, New Zealand; dan.cohen@outlook.co.nz (D.C.); whenshall@actrix.co.nz (W.H.); robin.macdiarmid@plantandfood.co.nz (R.M.M.); 2 The New Zealand Institute for Plant and Food Research Limited (PFR), Private Bag 1401, Havelock North 4157, New Zealand; vaughn.bell@plantandfood.co.nz; 3 Virology-Phytoplasmology Laboratory, Agroscope, 1260 Nyon, Switzerland; arnaud.blouin@agroscope.admin.ch; 4 The New Zealand Institute for Plant and Food Research Limited (PFR), P.O. Box 845, Blenheim 7240, New Zealand; dion.mundy@plantandfood.co.nz; 5 School of Biological Sciences, The University of Auckland, Private Bag 92019, Auckland 1142, New Zealand

**Keywords:** grapevine leafroll disease, grapevine leafroll-associated virus 3, visual symptoms identification, host–virus interaction

## Abstract

Grapevine leafroll disease (GLD) constrains wine production worldwide. In New Zealand, the main causal agent of GLD is grapevine leafroll-associated virus 3 (GLRaV-3). To control GLD, an integrated management program is used and includes removing (roguing) GLRaV-3-infected vines from the vineyard. The classical foliar symptoms from virus-infected red-berry cultivars are leaves with dark red intervein, green veins, and downward rolling of margins. Growers use these phenotypic cues to undertake visual symptom identification (VSI) for GLD. However, the influence of the known large genetic variation among GLRaV-3 isolates on the foliar symptoms from different grapevine cultivars remains undescribed, especially in cool-climate growing environments, such as New Zealand. Over three vintages (2015, 2016, and 2017), VSI for GLD was undertaken at three field sites in New Zealand (Auckland, Hawke’s Bay, and Marlborough), each including four cultivars (Merlot, Pinot noir, Sauvignon blanc, and Pinot gris) infected with three GLRaV-3 genotypes (Groups I, VI, and X) or GLRaV-3-uninfected control plants. Throughout this study, no visual symptoms were observed on white-berry cultivars infected with GLRaV-3. For red-berry cultivars, the greatest variability in observed foliar symptoms among regional study sites, cultivars, and GLRaV-3 genotypes was observed early in the growing season. In particular, Group X had significantly delayed symptom expression across all three sites compared with Groups I and VI. As the newly infected, young vines matured in years 2 and 3, the GLRaV-3 genotype, cultivar, region, and environmental conditions had minimal influence on the accuracy of VSI, with consistently high (>95%) within-vintage identification by the end of each vintage. The results from this study strongly support the use of VSI for the GLD management of red-berry cultivar grapevines, Merlot and Pinot noir, as a reliable and cost-effective tool against GLD.

## 1. Introduction

When plants become infected with a pathogen, they can display a range of symptoms, such as deformation, discoloration, and wilting, or death of organ parts, whole organs, or the entire plant. Symptom recognition and evaluation of its intensity is often employed in plant virology. For example, visual cues are the basis of the biological indexing process used to monitor regulated viruses in plant material for the trade or production of clean stock [1]. Furthermore, visual symptom identification (VSI) is widely used by grape growers to identify infected plants for removal [2,3].

Grapevine leafroll disease (GLD) is an economically significant viral disease that can lead to reduced vine vigor and longevity and reduced fruit yield and quality, which in turn negatively affects wine quality [4,5,6,7,8,9]. In New Zealand and worldwide, the leading causal agent for this disease is the grapevine leafroll-associated virus 3 (GLRaV-3). GLRaV-3 is the type member of the genus *Ampelovirus*, family *Closteroviridae* [10]. GLRaV-3 infection in grapevines has been shown to affect various aspects of the crop and grapevine health. A prominent visual GLRaV-3 foliar symptom is the eponymous downward rolling of the leaf margins and for red cultivar grapevines, the premature reddening of the leaves that starts as reddish spots that progressively spread to cover most, if not all, of the leaf surface, with the primary veins remaining green [5,11,12]. The leaves of some white-berry cultivars, such as Chardonnay, have been reported to turn yellow or chlorotic, but they do not exhibit the leaf reddening described for red-berry cultivars [5]. GLRaV-3 symptom development in the leaves of red cultivar grapevines has an asymptomatic period followed by a symptomatic phase (post-véraison) [4,6]. It is postulated that post-véraison, the phloem-limited GLRaV-3 causes an imbalance in the carbohydrate source-to-sink relationship, and this imbalance plays a key role in prematurely triggering the flavonoid biosynthesis [4,13,14,15]. The mechanism by which GLRaV-3 triggers the sugar imbalance has not been described.

This distinct foliar change in red cultivar grapevines is used as a cue for VSI of GLD in the New Zealand leafroll management strategy [2] and is becoming a more common tool in other countries for growers to identify virus infection for vine removal (roguing) [16]. VSI of red cultivars by a trained assessor has been demonstrated to be of high efficacy for four different red cultivars, with 99.9% of the 114,782 grapevines in one study agreeing with the laboratory enzyme-linked immunosorbent assay (ELISA) test [2]. It is important that the assessors who perform VSI are be well-trained to ensure the viral symptoms are not confused with abiotic stresses, such as nutrient deficiencies (e.g., magnesium and potassium) and girdling of canes, which often initiate flavonoid biosynthesis and generate similar reddening of leaves. GLRaV-3 has been reported to cause an upregulation of flavonoid biosynthesis genes in symptomatic leaves from red cultivar grapevines compared with the respective healthy leaves [13,17]. Conversely, the lack of the regulatory genes MYBA1 and MYBA2 in white cultivar grapevines likely leads to the lack of red pigment expression in their leaves [18,19,20].

To date, comparative studies regarding the impact of GLD on grapevines have produced conflicting results and differing interpretations [4,5,12,21]. Discrepancies are likely because of the varying biotic influences (e.g., virus genotype, cultivar, duration of infection, scion/rootstock, and combined infection with other viruses) and/or abiotic influences (e.g., climatic conditions, soil mineral content, and vine management practices). These complexities make it particularly difficult to gauge the possible impacts one virus has on the grapevine and berry/wine quality when comparing studies.

Genetic variation in plant virus populations can lead to differences in symptom development, as demonstrated by the well-studied citrus tristeza virus (CTV), another phloem-limited virus in the family *Closteroviridae*. CTV genotypes can cause different symptoms across different citrus species/cultivars, and different CTV genotypes can cause different symptoms in the same citrus species/cultivars [22]. The GLRaV-3 population worldwide shows substantially high genetic diversity, comprising at least eight different phylogenetic groups (Groups I, II, III, V, VI, VII, IX, and X) that can differ by more than 20% across the entire genome [23]. GLRaV-3 genotypes representative of Groups I to III, V, VI, and X have been identified in New Zealand commercial vineyards [24,25]. GLRaV-3 Groups I, VI, and X exhibit more than a 20% difference from each other based on the entire genome. To date, little research has been conducted to understand the effects of this high genetic diversity on symptom development [24,26,27,28,29,30,31,32,33,34]. However, results from a previous study suggest that VSI-based vine roguing may be biased toward severe strains and that the prominent distribution of GLRaV-3 Group X in the field may result from milder symptom expression (missed by the assessor) and/or more efficient vector transmission [26].

This study aimed to understand the effects of three distinct GLRaV-3 genotypes (Groups I, VI, and X) on the development of grapevine foliar symptoms and how they might influence the accuracy of VSI for GLD. Subsequent research measuring grapevine growth and berry composition from white- and red-berry cultivars in New Zealand conditions has been undertaken and will be reported separately. Here, we present new insights into the complex and intricate plant, virus, and environment interactions that underlie the host response to GLD and the impacts that GLRaV-3 genotypes have on VSI outcomes in New Zealand.

## 2. Materials and Methods

To investigate how three distinct GLRaV-3 genotypes influence foliar symptom development, we established plantings of four grapevine cultivars (*Vitis vinifera* Merlot, Pinot noir, Pinot gris, and Sauvignon blanc) on the same rootstock clone (3309 Couderc (3309C); *V. riparia × V. rupestris*) in three New Zealand winegrowing regions (Auckland, Hawke’s Bay, and Marlborough). These grapevines were purposefully and synchronously infected with the same source material (single infections each of Groups I, VI, and X). In addition, the effects of combinations of these three distinct GLRaV-3 genotypes (i.e., dual infections of Groups I and VI, Groups I and X, and Groups VI and X) on the leaf symptomology within infected grapevines were examined at the Auckland site, across all four cultivars.

### 2.1. Plant Material and Propagation

To establish the tri-regional trial sites, three key steps were completed: (i) selection and propagation of appropriate GLRaV-3-infected material, (ii) green grafting inoculations of selected GLRaV-3-infected material to four grapevine cultivars, and (iii) planting and maintenance of each field trial plot.

A preliminary screen of prospective grapevines displaying typical GLD foliar symptoms that could be used as a source of virus inoculum from New Zealand commercial vineyards was conducted by double antibody sandwich ELISA (DAS-ELISA) [35] and conventional reverse transcription polymerase chain reaction (RT-PCR) using GLRaV-3-specific primers as described previously [26]. Following this testing, cane material from selected vines with GLRaV-3 infections of interest were collected, self-rooted, potted, and grown in a glasshouse.

To confirm the virus population in the source plant material, the propagated glasshouse plants were screened by DAS-ELISA [35], conventional and real-time RT-PCR assays using GLRaV-3-specific primers [26,27], and high throughput sequencing (HTS) of the double-stranded RNA following a previously described protocol [36,37]. The following set of rules in regard to virus population, in order of priority, was used to select the most suitable source of propagated plant material for the establishment of the regional sites. The selected source material: (i) had to have the correct GLRaV-3 infection of interest and no other grapevine leafroll virus or vitivirus, (ii) had to have the presence of grapevine rupestris stem pitting-associated virus (GRSPaV) was accepted, and (iii) must not have grapevine fleck virus. Refer to Table S1 for HTS results from source plant material screening and plant section.

Green cane material from the selected glasshouse GLRaV-3-source grapevines (single-and dual-infected) were green-grafted on top of the potted grapevines certified as GLRaV-3-free (potted plants were sourced from and grafts performed by Riversun Nursery Limited, Gisborne; Table S2 presents green graft success rates). The potted grapevines consisted of four scion cultivars imported from Entav-INRA: Merlot 181, Pinot noir 777, Pinot gris 52, and Sauvignon blanc 376. To generate dual GLRaV-3 Groups I and VI infected grapevines, after 8 weeks, a second green bud from plant material positive for Group VI was grafted on the 3309C rootstock of grapevines with a successful first graft with plant material positive for Group I. Additionally, after the initial planting in 2014, we sought to resolve a shortfall in grapevines, with additional green grafting performed and planted in 2015 (Table S3). For these additional graft events, the grapevines were tested by DAS-ELISA [35] and subsequently by quantitative real-time RT-PCR (RT-qPCR) using generic and variant specific primer sets [26,27], and high-resolution melting curve (HRM) analyses (Section 2.6) were conducted prior to planting at the Auckland field plot.

### 2.2. Site Location and Study Configuration

A single trial site was established in each of the three wine grape regions in New Zealand: Auckland (36°49′22.03″ S, 174°28′35.13″ E), Hawke’s Bay (39°39′13.56″ S, 176°52′34.74″ E), and Marlborough (41°29′26.26″ S, 173°57′20.06″ E). At each site, four grapevine cultivars (*V. vinifera* Merlot, Pinot noir, Pinot gris, and Sauvignon blanc) infected with single infections of the GLRaV-3 genotype representative of Groups I, VI, and X [23] and associated healthy controls for each cultivar were planted. In addition, at the Auckland site, *V. vinifera* Merlot, Pinot noir, Pinot gris, and Sauvignon blanc grapevines were infected with two GLRaV-3 genotypes (Groups I and VI, Groups I and X, and Groups VI and X), and their respective healthy controls were planted. Twenty biological replicates for each virus treatment and cultivar at each site were prepared. However, grapevine mortality after planting and failure to inoculate the GLRaV-3 genotype resulted in some treatments at each site varying between 16 and 20 biological replicates, with the exception of Pinot gris inoculated with Groups I and VI where only 13 plants were obtained (Tables S3 and S4).

To ensure the grapevines were spread evenly throughout each of the sites and to control for possible terrain differences, a planting plan based on the Williams Design [38] was implemented. Each bay (an area between two trellis posts along a vine row) contained five vines of the same treatment (GLRaV-3-infected or healthy control) and a single healthy grapevine (of the same cultivar) that acted as a “buffer grapevine”. The “buffer grapevines” were regularly visually assessed, and ELISA tested to determine if there was secondary mealybug (insect vectors of GLRaV-3) spread occurring within the field plot.

### 2.3. Planting and Maintenance of Each Field Trial Plot

In late January to early February of 2014, a total of 1,432 grapevines (i.e., grapevines that showed successful green grafts with growth of grafted shoots (single and dual GLRaV-3 infections), associated healthy grapevines, and “buffer grapevines”) were planted at the three sites. Early in vintage 2015 (i.e., December 2014), an additional 107 grapevines were planted across the three regional sites. These replaced grapevines that did not survive the first planting or grapevines with additional bud grafts to fulfil the replicate numbers for the dual GLRaV-3-infected grapevines. After the grapevines were planted, the grafted shoot (i.e., the source of GLRaV-3 inoculum) was removed from the grapevines to ensure only shoots from the intended scion grew.

Over the three years, grapevines at each field plot were maintained in accordance with New Zealand wine industry standards. This involved standard drip irrigation, adopting industry fungicide and insecticide spray regime recommendations, and standard viticultural practices for 1-cane laid grapevines. To prepare grapevines for vintage 2015, all vines were pruned to two buds (to encourage strong growth of a single shoot to be laid along the fruiting wire). To prepare grapevines for vintages 2016 and 2017, grapevine pruning options were as follows: grapevines that did not reach the fruiting wire or had poor cane development (i.e., small cane diameter) were pruned to two buds; for grapevines that reached the fruiting wire, a single cane was laid on the wire and pruned to eight buds.

To ensure continuity of the GLRaV-3 status among the virus-infected, healthy control, and buffer vines, grapevine leaf or cane material was sampled annually and tested by DAS-ELISA [35]. Selected samples were also tested by RT-qPCR [27].

### 2.4. Climate Data

Throughout this project, weather data were collected for the Hawke’s Bay and Marlborough sites from long-established weather stations that were either on-site (Hawke’s Bay) or less than 2 km from the site (Marlborough). In Auckland, an on-site weather station collected temperature, relative humidity, and rainfall data on an hourly basis from 18 November 2015. Data from the Auckland site were manually acquired from the weather station; data from the Hawke’s Bay and Marlborough weather stations were acquired through the MetWatch Online website (http://www.hortplus.metwatch.co.nz/index.php; assessed on 2 September 2021). The hourly data were summarized on a monthly basis, and general indices were calculated.

To gain an indication of annual climatic variation at each site and the extent of the climatic difference among sites, the mean temperature of the warmest month (MTWM) (°C), mean temperature of the coldest month (MTCM) (°C), and annual rainfall (mm) were calculated. In addition, commonly used methods to assess site quality for growing grapevines and the particular cultivar(s) to be grown at a site, such as growing degree days (GDDs) (between 1 September and 30 April, 10 °C base temperature) and latitude temperature index (LTI) [39], were assessed from available data.

### 2.5. Visual Symptom Identification (VSI)

**Figure 1:**
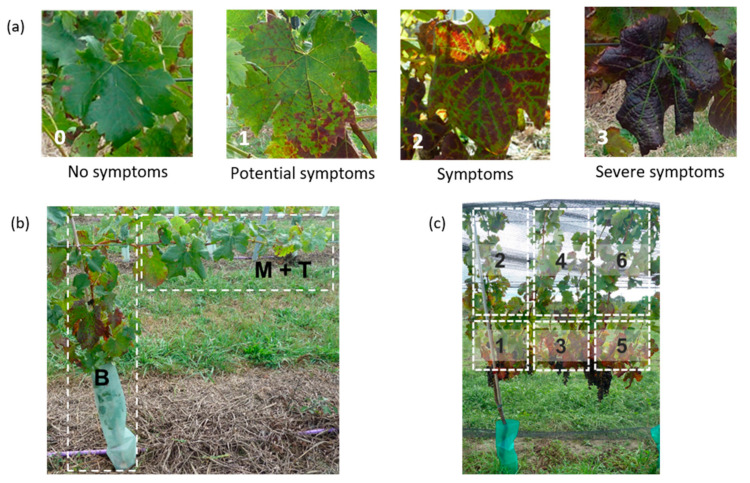
Grapevine leafroll disease (GLD) visual symptom identification (VSI) assessment criteria for all sites during vintages 2015, 2016, and 2017. (**a**) Symptom scale (0 to 3) for GLD foliar symptoms on red-berry grapevine leaves. The images were used as visual comparisons to enable standardization of VSI scoring at all three regional sites. (**b**) The young grapevine canopy of red-berry cultivars (assessed in the 2015 vintage) was divided into two zones: base (B) and middle to top (M + T) canopy zones, as indicated by the white dotted boxes. (**c**) In vintages 2016 and 2017, the mature grapevine canopy of red-berry cultivars was divided into six zones for VSI, as indicated by the white dotted boxes. All photographs are of *Vitis vinifera* Merlot.

Southern Hemisphere growing seasons (September to April) are hereafter referred to as vintages for 2015, 2016, and 2017. During each of the three vintages reported, visual assessments of foliar symptoms in red and white cultivar grapevines were performed by the same VSI-trained researchers stationed at each field site [2]. The visual symptoms on grapevines were monitored fortnightly to monthly, as required, from late December/early January to mid-April. For red-berry grapevines, foliar symptoms were assessed based on a symptom score range from 0 (no symptoms) to 3 (severe symptoms) (Figure 1a); the foliar symptoms in white-berry grapevines were assessed based on a “yes” or “no” criterion. A red-berry grapevine was visually identified as GLRaV-3-positive when vines were assessed as a “2” or “3” based on the symptom assessment scale. The locations of observed foliar symptoms were also recorded. In the 2015 vintage, the young canopy was divided into two zones: (i) the base zone and (ii) the middle to top zone of the growing shoot (Figure 1b). At the Auckland and Hawke’s Bay sites, as the vines matured in vintages 2016 and 2017, the canopy was sectioned conceptually into six zones (three zones spanning the length of the fruiting wire and the other three directly above, comprising the upper canopy) (Figure 1c).

### 2.6. Quantitative Real-Time RT-PCR (RT-qPCR) and High-Resolution Melting Curve (HRM) Analysis

Approximately 100 mg of plant tissue was used for total RNA extractions, using a modified cetyltrimethylammonium bromide (CTAB) extraction method [40]. In addition to the RT-PCR and real-time RT-PCR assays using GLRaV-3-specific primers [26,27], a high-resolution melting curve (HRM) analysis was performed to assist with confirming the GLRaV-3 genotype infection status of single- and mixed-infected grapevines. Reverse transcription was performed using the designed generic virus-specific reverse primer (GLR3-GEN-9730R: 5′-CCTTCAGGACCTAGCACTTTCAGCG-3′) and the standard manufacturer’s protocol (Invitrogen, Carlsbad, CA, USA). The PCR amplification and high-resolution melting curve analysis were carried out in 10 µL reactions using the HOT FIREPol^®^ EvaGreen^®^ HRM Mix (Solis BioDyne, Tartu, Estonia) and performed in an Illumina Eco™ Real-Time PCR System. Each reaction also contained 200–400 ng of total RNA extract and 200 nM of each forward (GLR3-GEN-9558F: 5′-GTCTTTGGTGGACGACGGGAG-3′) and reverse (GLR3-GEN-9730R) primer. Thermocycling conditions were 95 °C for 5 min, 40 cycles of 95 °C for 10 s, annealing at 60 °C for 20 s, and extension at 72 °C for 20 s, and the final high-resolution melt curve analysis was 95 °C for 15 s, 55 °C for 15 s, and 95 °C for 15 s.

## 3. Results

### 3.1. Site Climate Based on Air Temperature and Rainfall

Over the data collection time, at each site, the mean monthly temperatures (MTWMs and MTCMs) were similar among vintages (Table 1). The MTWM was similar across the three sites, while the Auckland site had a higher MTCM than the other two sites. The annual rainfall increased gradually over the 3-year study for all three sites. Auckland had the highest annual rainfall among the three sites, with at least 182% more rainfall than Hawke’s Bay and Marlborough (ranged between 182 and 259%). The Hawke’s Bay site generally received more rainfall than the Marlborough site (112–159% more). The GDDs were generally similar across the three sites, with the exception of the 2017 vintage where the Hawke’s Bay site had significantly higher GDDs than the other two sites. Consistently, Auckland and Hawke’s Bay had a higher LTI than the Marlborough site across all three vintages.

### 3.2. Visual Symptom Identification (VSI) of Grapevines for Grapevine Leafroll Disease (GLD)

Over the three years of VSI for GLD foliar symptoms at the three regional sites, GLD was observed only among the red cultivars, Merlot and Pinot noir grapevines, known to be infected with GLRaV-3 (single or dual GLRaV-3 genotype-infected). No specific foliar symptoms were observed among (a) the white cultivars (Pinot gris and Sauvignon blanc) infected with GLRaV-3 (single or dual GLRaV-3-infected), (b) among the GLRaV-3-negative (virus-free) red cultivar grapevines, or (c) among the buffer grapevines. The absence of GLRaV-3 in the buffer and the nongraft-inoculated vines was confirmed by DAS-ELISA and RT-qPCR. Thus, there was no evidence of a vector-mediated spread of GLRaV-3 at any regional site.

#### 3.2.1. Visual Symptom Identification of Grapevines Infected with One GLRaV-3 Genotype

**Figure 2:**
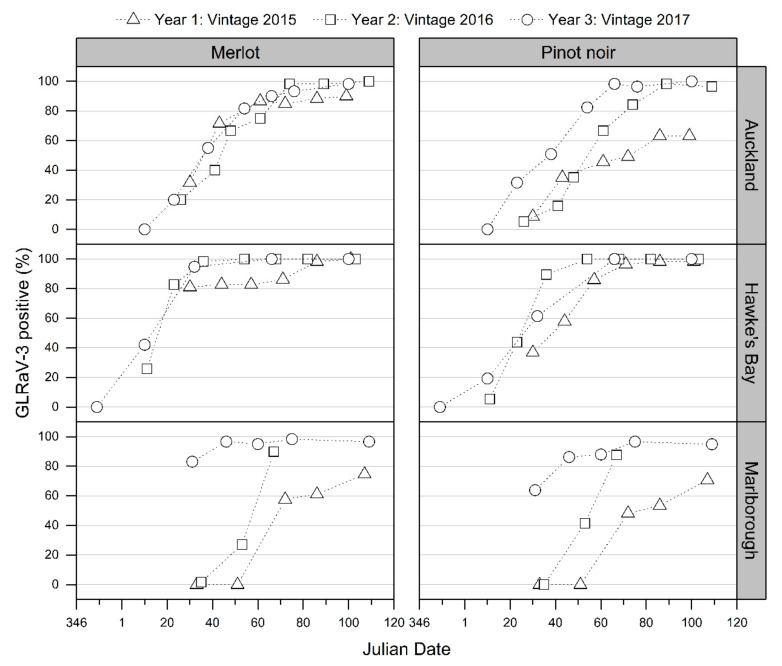
Visual symptom identification of grapevine leafroll-associated virus 3 (GLRaV-3) in the field trial across three regional locations (Auckland, Hawke’s Bay, and Marlborough) and two red-berry cultivars (Merlot and Pinot noir) for vintage 2015 (triangle points), 2016 (square points), and 2017 (circle points). Data are percentages of Merlot and Pinot noir vines (left and right, respectively) singly infected GLRaV-3 (irrespective of genotype) that were visually identified as GLRaV-3-positive based on distinctive foliar changes (i.e., assessed as a “2” or “3” based on the symptom assessment scale). Over the three vintages, no visual symptoms were observed on any white-berry cultivars (Pinot gris and Sauvignon blanc) at any of the regional locations; therefore the results are not displayed.

Over the three vintages at all sites, positive VSI, and thereby the VSI accuracy, increased gradually over each vintage (Figure 2). The lowest proportions of positively identified GLRaV-3-infected red cultivar grapevines (i.e., lowest VSI accuracy) were at early inspection time points, and the highest and most consistent identifications of GLRaV-3-infected grapevines (i.e., highest and most consistent VSI accuracy) were late in each vintage (mid-March–April, from Julian date 70 onward) (Figure 2).

At all sites, a noticeably higher proportion of red cultivar grapevines showed foliar symptoms earlier in vintage 2017 (year 3 of visual assessments) than in assessments undertaken in the 2015 vintage (year 1 of visual assessments) and 2016 vintage (year 2 of visual assessments). In addition, GLD symptoms were generally observed earlier in each vintage among the Merlot grapevines infected with GLRaV-3 than among infected Pinot noir vines (Figure 2).

In each vintage, regional differences in the proportion of GLRaV-3-infected Merlot and Pinot noir grapevines positively identified with GLD foliar symptoms were observed during early VSI time points (Figure 2). For example, at the first assessment in February of the 2016 vintage (Julian days 35, 36, and 41 for the Marlborough, Hawke’s Bay, and Auckland assessments, respectively), only 1 out of 59 Merlot GLRaV-3-infected grapevines were observed to have GLD symptoms at the Marlborough site compared with 40% (24 out of 60) and 98% (57 out of 58) at the Auckland and Hawke’s Bay sites, respectively. This difference could not be fully explained by variable VSI assessor capability. During the 2016 vintage (Julian day 48), both the Auckland and Hawke’s Bay assessors inspected the grapevines at the Auckland site independently of one another. The Hawke’s Bay assessor positively identified 68% of the Merlot (41 out of 60) and 42% of the Pinot noir (24 out of 57) GLRaV-3-infected grapevines; the Auckland assessor visually diagnosed 67% (40 out of 60) and 35% (20 out of 57) of the Merlot and Pinot noir GLRaV-3-infected grapevines, respectively.

#### 3.2.2. GLD Symptoms Based on Virus Genotype and Spread of Symptoms over the Canopy

**Figure 3:**
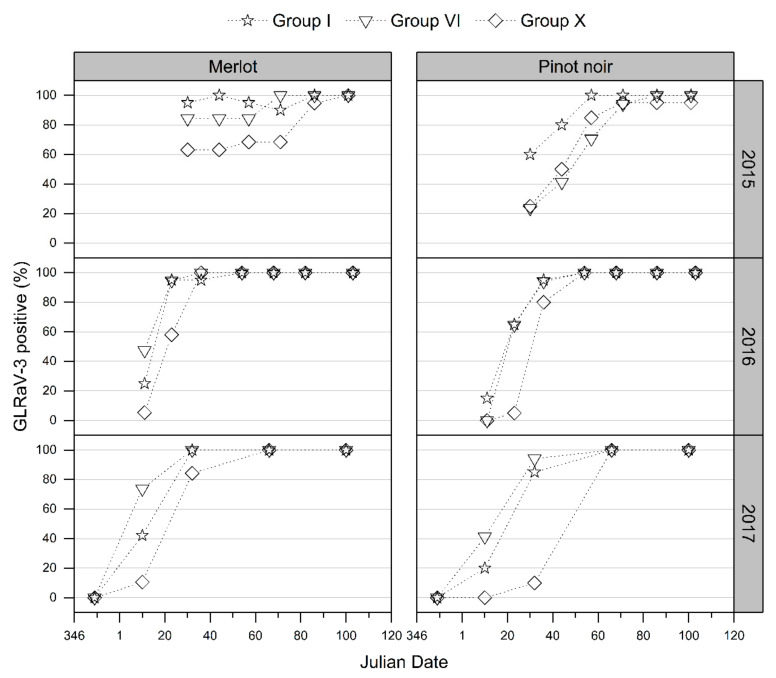
Visual identification of grapevine leafroll-associated virus 3 (GLRaV-3) genotypes at the Hawke’s Bay site for the red-berry cultivars Merlot (left panel) and Pinot noir (right panel) in vintages 2015, 2016, and 2017. Generally, in all vintages, delayed symptom expression was observed for Merlot and Pinot noir (especially in vintages 2016, 2017) infected with Group X (diamond points) relative to Group I (star points) and Group VI (triangle points) virus infections. This reduced the overall percentage of GLRaV-3-infected vines positively identified by foliar symptoms, with the exception of vintage 2015, where the percentage of Group X-infected Pinot noir vines positively identified for disease based on foliar symptoms was greater than that for Group VI in the same cultivar.

**Figure 4:**
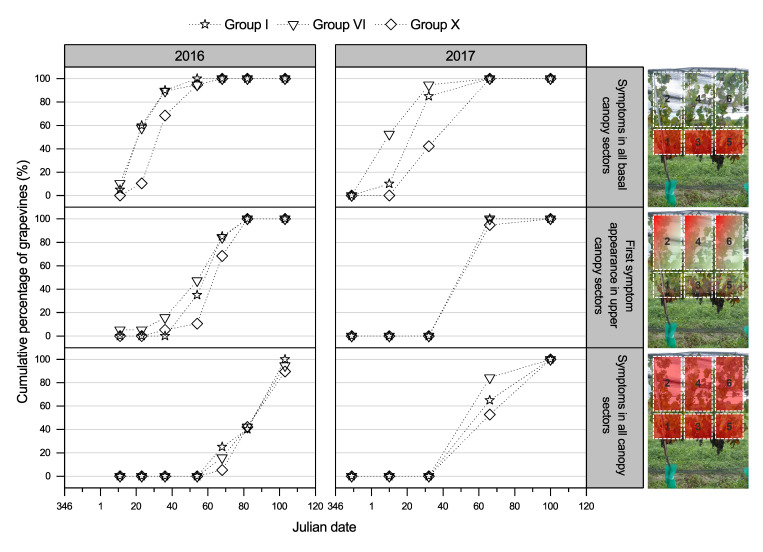
Foliar symptoms of the grapevine leafroll-associated virus 3 (GLRaV-3) Group X genotype appeared later than the Group I and IV genotypes symptoms from infected Merlot grapevines, as recorded in the Hawke’s Bay study site during vintages 2016 (left panel) and 2017 (right panel). For all genotypes, the expression of foliar symptoms moved from the laid cordon (base of the canopy) upward, as the growing season progressed. Presented are the cumulative percentages of grapevines infected with genotypes Group I (star points), Group VI (triangle points), and Group X (diamond points) with observable leafroll symptoms in all basal canopy sectors (demarcated as Sections 1, 3, and 5), the first symptom appearance in the upper canopy sectors (demarcated as Sections 2, 4 and 6), and symptoms in all canopy sectors (demarcated Sections 1 to 6).

At all three regional study sites, the foliar symptoms for both Merlot and Pinot noir cultivar grapevines infected with the Group I or VI genotypes expressed symptoms earlier in the vintage than Group X-infected grapevines (Figure 3 displays results from the Hawke’s Bay site; results from Auckland and Marlborough are presented in Figures S1 and S2). For all GLRaV-3 genotypes, a similar trend for GLD vertical progression of symptom expression across the canopy was observed during vintages 2016 and 2017. Initially, symptoms were observed at the basal portions of the canopy (canopy region nearest the laid cane, referred to as Sections 1, 3, and 5 in this study), but as each growing season advanced, viral symptoms were progressively observed in the upper canopy (Sections 2, 4, and 6), with overall symptom expression more consistent throughout the canopy late in the vintage (Figure 4, Merlot grapevines at the Hawke’s Bay site; Figure S3, Pinot noir grapevines at the Hawke’s Bay site; and Figures S4 and S5, Merlot and Pinot noir grapevines at the Auckland site). As with the delayed assessment of symptomatic Group X-infected grapevines, the spread of visible foliar symptoms from the basal to the upper sections of the grapevine canopy was also slower in Group X-infected Merlot and Pinot noir grapevines relative to the other virus genotypes in this study. No apparent difference in the visible foliar symptoms expressed by the different GLRaV-3 genotypes horizontally across the canopies was observed (Figures S6 and S7). A similar proportion of Merlot and Pinot noir grapevines showed GLD symptoms in shoots closest to the trunk compared with shoots furthest away from the trunk at each time point over each growing season (Figures S6 and S7).

### 3.3. Visual Symptom Identification of Grapevines Infected with Two GLRaV-3 Genotypes

Similar to the grapevines infected with a single GLRaV-3 genotype, the VSI accuracy of dual GLRaV-3-infected grapevines increased gradually over each vintage, and late in each vintage the most consistent detection of GLD foliar symptoms was observed (Figure S8). This gradual increase in the proportion of positively identified Merlot and Pinot noir grapevines infected with two GLRaV-3 genotypes matched those of grapevines infected with one GLRaV-3 genotype in vintages 2016 and 2017 (years 2 and 3) (Figure S8). The exception was year 1 (vintage 2015), where a lower proportion of Merlot and Pinot noir vines with dual GLRaV-3 infections showed foliar symptoms compared with grapevines containing a single GLRaV-3 genotype, likely because some of the double-inoculated vines were produced later.

**Figure 5:**
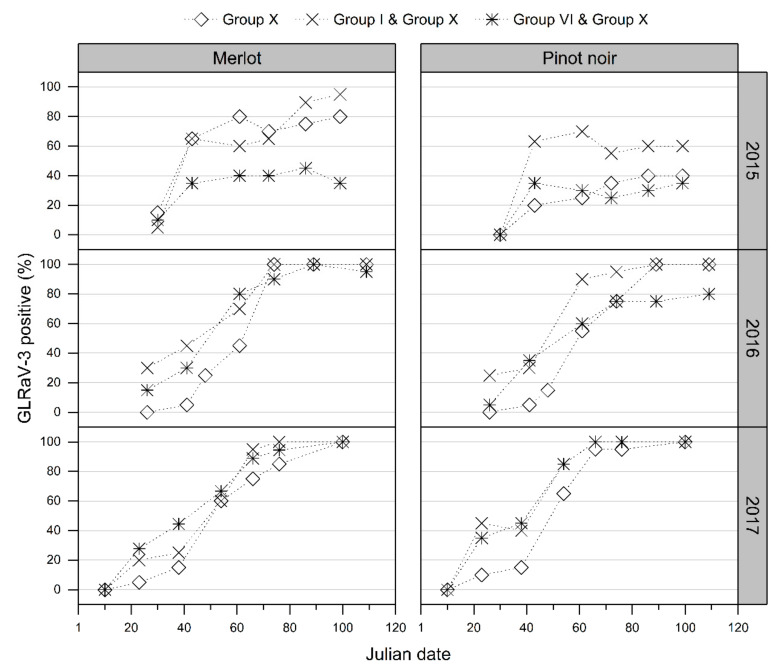
Visual identification of grapevine leafroll-associated virus 3 (GLRaV-3) from Merlot (left panel) and Pinot noir (right panel) grapevines infected with only the GLRaV-3 Group X genotype (diamond points) and with Group X in a dual infection with Group I (“X” points) or Group VI (“
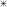
” points) genotype at the Auckland site, for the vintages 2015, 2016, and 2017.

Differences between grapevines infected with one GLRaV-3 genotype and their counterpart grapevines infected with the same genotype in a coinfection with another GLRaV-3 genotype were often observed at early VSI time points in each vintage (Figure 5 and Figures S9 and S10). Notably, lower proportions of Merlot grapevines with a coinfection of the Group I and X genotypes were consistently observed with leafroll symptoms at the early growing season assessment time points (between Julian days 30 and 60) compared with proportions of Merlot grapevines infected singularly with Group I or coinfected with Groups I and VI) (Figure S9). Moreover, generally fewer red cultivar grapevines with Group VI coinfected with Groups I or X were observed with leafroll symptoms compared with grapevines infected with Group VI only. In particular, the visual symptom expression of GLRaV-3 in Merlot and Pinot noir grapevines was less pronounced when Group VI was coinfected with Group X in the 2017 vintage (Figure S10). For example, at Julian day 38, 44% Merlot and 45% Pinot noir grapevines with dual Group VI and X infection showed GLD symptoms, compared with 70% Merlot and 82% Pinot noir grapevines infected with only the GLRaV-3 Group VI genotype. Conversely, for two consecutive years (vintages 2016 and 2017), at early vintage time points, a greater proportion of Merlot and Pinot noir grapevines coinfected with Group X and either Group I or Group VI was observed with leafroll symptoms compared with the respective grapevines infected with Group X only (Figure 5).

## 4. Discussion

Studying the disease etiology of phloem-limited viruses, such as GLRaV-3, that infect perennial plants is difficult in the natural environment without access to an infectious viral clone that infects the natural host systemically. In most GLRaV-3 studies, important factors, such as virus genotype(s), time of infection, duration of infection, geographic location, presence of other viruses, and/or scion/rootstock, are not considered [4,5,12,21]. This lack of comparative rigor has probably led to discrepant findings between studies. To our knowledge, this study is the first field trial that used purposefully infected different grapevine cultivars with different GLRaV-3 genotypes by grafting and planting grapevines in three geographically distinct regions and that assessed disease expression over multiple vintages. Here, we discuss critically how the physiological data presented in this study reflect existing commentaries and hypotheses for GLD foliar symptom development; we describe new insights into the importance of plant–virus interactions for disease expression and discuss the practical implications of virus genotypes on the utility of VSI for GLD management in the vineyard.

### 4.1. Véraison Coincides with GLD Foliar Symptoms in Red-Berry Grapevines

This study supports the current GLRaV-3 hypothesis that GLD foliar symptoms of red cultivar grapevines have two phases: a pre-véraison asymptomatic phase and a postvéraison symptomatic phase [4,6]. After 3 years of observations, typical leafroll foliar symptoms in Merlot and Pinot noir red-berry cultivars were only apparent post-véraison (after mid-January in New Zealand) at all three trial sites (Figure 2). There were no asymptomatic vines from vines known to be infected with GLRaV-3. Even after 8 years from the initial infection, no foliar symptoms were observed prior to véraison in the 2021 vintage (K. Chooi personal observation; data not shown).

In vintages 2016 and 2017, in particular, a higher proportion of grapevines was observed with foliar symptoms earlier in the season, which supports previous observations that older infections of GLRaV-3 are more likely to appear earlier in the growing season [2]. Based on our results and current knowledge, there appears to be a strong temporal link between GLD foliar symptoms caused by GLRaV-3 and carbohydrate movement within the grapevines in New Zealand. At véraison, berries undergo a rapid size increase accompanied by turgor loss, softening, sugar accumulation, and color accumulation in red-berry cultivars, and xylem hydraulic conductance decreases [41,42]. At the same time, the main water transport pathway changes from xylem to phloem [43,44,45], and sugar transport shifts from the symplastic to apoplastic pathway [46]. Is it possible the phloem-limited virus influences this complex network of interactions and signals linked to véraison?

Notably, after 3 years of observations in New Zealand, GLRaV-3-infected Pinot gris and Sauvignon blanc white-berry cultivars lacked any foliar symptoms, such as chlorosis and leaf rolling. This lack of foliar symptoms contradicts other observations described overseas, mostly on Chardonnay [5] and may in part be caused by additional factors, such as cultivar/clone, environment, and the role of mixed viral infections. Although no foliar symptoms were observed in these white cultivars in this study, more research is required to understand whether GLRaV-3 influences other grapevine aspects. Subsequent research measuring grapevine growth and berry composition from white- and red-berry cultivars in New Zealand conditions has been undertaken and will be reported separately.

### 4.2. GLRaV-3 Genotypes, Foliar Symptom Development, and Potential Plant–Virus Interactions

Collected climate data provide some insights into the intra- and inter-site variability in this study. Based on the grape-ripening capacity predictions using GDDs, all three sites had similar ‘degree days’ or ‘heat units’ and therefore similar potential vine growth and development (Table 1). With an LTI of less than 380, the Marlborough site could be considered a warm climate region, where key cultivars, such as Merlot and Sauvignon blanc, are often grown [39]. In contrast, the Auckland and Hawke’s Bay sites would be considered as warm to hot climate regions (LTI greater than 380). Additionally, the three field sites had notable differences in rainfall. Climatic and environmental aspects, such as frost potential, wind, site elevation, and soil type, were not assessed.

Regardless of the environmental conditions measured at the three regional sites, all red-berry grapevines showed foliar symptoms. Foliar symptom differences observed among sites were negligible and far less important than those measured among treatments (genotypes), which confirms in this case, that the genotype overcomes the environmental factors. Foliar symptoms were consistently observed earlier in Group I-infected Merlot and Pinot noir grapevines than in Group X-infected grapevines throughout this study (Figure 3 and Figures S1 and S2). The difference in symptom expression among genotypes was most prominent early in the vintage at the lower canopy sections and at the upper portions of the canopy later in the vintage (Figure 4 and Figures S3–S5). This consistent observation of differential onset of leaf reddening in grapevines infected with different GLRaV-3 genotypes implies that GLRaV-3 and its genotype directly influence the timing and apparent severity of foliar symptoms. The expression of leaf reddening in GLRaV-3-infected grapevines is assumed to be caused by anthocyanin synthesis [13,17]; therefore, it is reasonable to assume that the different timing of the onset of leaf reddening observed may be caused by an alteration in the anthocyanin pathway.

Plant viruses are known to interact and influence an array of plant miRNAs, plant pathways, and plant proteins to cause observable disease symptoms [47,48,49,50]. Phloem-limited virus CTV and sugarcane yellow leaf virus have shown that they directly modulate both sugar/carbohydrate and ROS synthesis and movements in plants [49,51,52], while potyvirus and a bromoviridae virus have been demonstrated to modulate anthocyanin biosynthesis through direct interaction of viral suppressors of RNA silencing (VSRs) encoded by these viruses with the anthocyanin regulatory pathway [47,48]. Higher starch and sugar contents [13,14,15] and upregulation of genes associated with anthocyanin biosynthesis [13,17,53] in symptomatic leaves from GLRaV-3-infected red-berry cultivars compared with leaves of a similar age from healthy grapevines have been reported. Moreover, comparative studies of a known GLRaV-3 VSR protein, p19.7, encoded by different genotypes using in vitro laboratory assays demonstrated variable VSR activity [28,54].

Rapid cell-to-cell and systemic virus movement may result in earlier and more apparent symptom expression as shown for a modified tomato bushy stunt virus (TBSV) coat protein (CP) [55,56]. Virion-associated proteins considered to play a role in virus movement encoded by GLRaV-3 are highly divergent among the three genotypes used in this study (between 5 and 22% amino acid differences). Differences in the N-terminus of the GLRaV-3 CP likely alter the exposed structure(s) on the virion surface [24,35]. GLRaV-3 genotypes have been previously reported to be unevenly distributed and at different virus titers in plants, particularly the Group X genotype that was inconsistently detected and at a lower titer using molecular assays along a grapevine shoot compared with the detection of Groups I and VI [27,57]. Collectively, the reported uneven GLRaV-3 genotype distribution and variable GLRaV-3 VSR strengths support the hypothesis presented in the current study that GLRaV-3 proteins influence host proteins and pathways with varying potencies, which lead to different foliar symptoms through the season. Further studies using yeast two-hybrid and bimolecular fluorescence complementation systems may elucidate the specific host–virus protein interactions. Use of host proteins from different cultivars will provide additional insights into variable cultivar responses to virus. In this study, the Merlot GLRaV-3-infected grapevines developed foliar symptoms faster than Pinot noir GLRaV-3-infected grapevines (Figure 2). Targeted in-vitro experiments, such as the overexpression of VSR proteins in known plant systems where influences on the anthocyanin biosynthesis can be measured, could also help elucidate the direct role of GLRaV-3 VSRs.

### 4.3. Dual Infections of Different GLRaV-3 Genotypes Influenced the Onset of GLD Foliar Symptom Expression Compared with Single Infections of the Same GLRaV-3 Genotype

Generally, early in the vintage, the grapevines co-infected by Group X along with Groups I or VI produced an intermediate response compared to the symptoms observed from a single Group X GLRaV-3 infection and those from Group I or VI single infections (Figure 5 and Figures S9 and S10). The observed variable symptom expression that was dependent on the type of GLRaV-3 genotype(s) further supports the hypothesis that GLRaV-3 specifically interacts with and influences plant host pathways and proteins. In particular, Groups I and VI caused greater detrimental effects to the plant foliage than Group X. This result also raises the questions: Do the Group I and VI genotypes have greater fitness with intra-plant movement and plant defense suppression? Are Group I and VI genotypes able to occupy and initiate plant stress responses in more plant cells, resulting in faster leaf reddening? Are there synergistic/complementary virus–virus interactions among the GLRaV-3 genotypes that aid the accumulation, movement, and/or virulence of the Group X genotype?

### 4.4. Visual Symptom Identification for GLD Foliar Symptoms in Red-Berry Grapevines Is an Effective Method for GLD Management

VSI by trained staff for GLD foliar symptoms in red-berry grapevines has been demonstrated to be an effective GLD management strategy to cost-effectively identify diseased grapevines for removal and planting replacement grapevines [2]. Since the GLRaV-3 genotype was not taken into account by Bell et al. [2], the question remained whether the GLRaV-3 genotype affects the expression and timing of symptom development and in turn the efficacy of VSI. Our results, which include data from the two main grape-growing regions of New Zealand (Hawke’s Bay and Marlborough) strongly support the use of VSI by trained assessors to identify GLD in red-berry cultivars. This result was independent of the GLRaV-3 genotype tested. Under our conditions, Group X-infected grapevines expressed foliar symptoms, which suggest that the previously reported prominent distribution of the Group X in a commercial vineyard [26] is more likely because of different transmissibility rates by mealybugs rather than mild symptom expression influencing VSI. We also confirmed the absence of any foliar symptoms among grapevines in either of the two white cultivars used in this study. These observations across multiple years reiterated the need for serological or molecular testing to identify virus-infected grapevines reliably. We note, however, the additional cost of this testing may be a barrier to many growers assessing virus in white cultivars, which as a virus reservoir has potential to threaten other vine plantings in the vineyard.

For at least three dissimilar genotypes that represent the genetic extremes known to occur in New Zealand, there is unlikely to be a GLRaV-3 genotype impact on the overall detection of GLD in infected Merlot and Pinot noir grapevines if the VSI is performed as recommended [2,58]. Visual assessments undertaken later in a vintage (before leaf fall) offer the greatest prospect to identify a high percentage of symptomatic grapevines, particularly newly infected grapevines, grapevines that have been leaf plucked (removing leaves from the base of the canopy most likely to exhibit GLD symptoms), and/or grapevines infected with genotypes that express late foliar symptoms, i.e., the Group X genotype. In the future, there will need to be a better understanding of the compounding effects such as coinfection with other grapevine viruses. An example is a dual GLRaV-1 infection that may have fewer foliar symptoms that are less distinctive than GLRaV-3, thereby potentially reducing the efficacy of VSI assessments.

## 5. Conclusions

In the absence of an infectious GLRaV-3 clone that can systemically infect grapevines, this study demonstrated that GLRaV-3 causes the distinctive foliar changes in red-berry cultivar grapevines. This is the first study to describe foliar symptoms monitored across the canopy over a season and over multiple vintages. Regardless of the environmental conditions, the foliar symptoms caused by the GLRaV-3 genotype were indistinguishable from one another apart from the onset and general appearance of GLD symptoms across the canopy. Foliar symptoms from Group I-infected Merlot and Pinot noir vines were observed consistently earlier than Group X-infected grapevines. There are indications the virus genotypes can influence the timing and severity of symptom expression when coinfected in the same grapevine. GLRaV-3 did not cause distinctive foliar changes in the white-berry cultivars included in this study. It will be of particular interest to understand whether the different GLD foliar symptoms caused by the distinct genotypes in red-berry cultivar grapevines or lack of foliar symptoms in white-berry cultivar grapevines are also reflected in other aspects, such as plant physiology, including growth and berry yield and quality, and in subsequent wine quality.

## Figures and Tables

**Table 1:** Climate data from the three grape-growing sites collected in vintages 2015, 2016, and 2017, where possible. Mean temperature of the warmest month (MTWM) (°C), Mean temperature of the coldest month (MTCM) (°C), Annual rainfall (mm), Growing Degree Days (GDDs) (°C), and Latitude temperature index (LTI).

	Auckland	Hawke’s Bay	Marlborough
2015 vintage			
MTWM (°C)	n/a	18.4	18.8
MTCM (°C)	n/a	7.9	7.4
Annual rainfall (mm)	n/a	607.8	381.6
GDDs	n/a	1357.3	1367.2
LTI	n/a	374.4	347.8
2016 vintage			
MTWM (°C)	19.9	20.1	20.0
MTCM (°C)	10.3	8.8	8.3
Annual rainfall (mm)	1276	661.9	590.8
GDDs (from 18 November 2015)	1277.8	1183.6	1167.6
GDDs (complete vintage)	n/a	1357.3	1386.5
LTI	461.1	409.0	370
2017 vintage			
MTWM (°C)	18.2	19.7	18.6
MTCM (°C)	10.2	8.0	7.9
Annual rainfall (mm)	1538 ^#^	846.6	591.4
GDDs	1405.6	1614.1	1368
LTI	431.0	400.9	344.1

n/a, not applicable as data were not available because the weather station at the Auckland field site was only installed on 18 November 2015. ^#^ Missing rainfall data from the weather station at the Auckland field trial site were supplemented with data from weather station located in Mt Albert, Auckland (~24 km away).

## Supplementary Materials

The following supporting information can be downloaded at: https://www.mdpi.com/article/10.3390/v14071348/s1, Tables S1–S4, Figures S1–S10. Table S1: Detection of grapevine leafroll-associated virus 3 and other grapevine viruses and/or viroids by high throughput sequencing (HTS) in plant material from source plants used. Table S2: The success rate of the green-graft inoculations for each of the grapevine leafroll-associated virus 3 (GLRaV-3) infection types and grapevine cultivars. Table S3: Total number of biological replicates for each dual grapevine leafroll-associated virus 3 genotype infection treatment and cultivar planted at the Auckland field trial site. Grapevines were planted at the Auckland location across two vintages, 2014 and 2015. Table S4: Total number of biological replicates (Rep.), number of vines tested negative for virus after two consecutive years of laboratory testing (Neg.), and number of missing vines (Missing) for each treatment and cultivar in the Auckland, Hawke’s Bay, and Marlborough grapevine leafroll-associated virus 3 (GLRaV-3) field trial sites. Figure S1: Visual identification of grapevine leafroll associated virus 3 (GLRaV-3) genotypes at the Auckland site for the red-berry cultivars Merlot (left panel) and Pinot noir (right panel), in vintages 2015, 2016, and 2017. Generally, in all vintages, delayed symptom expression was observed for Merlot and Pinot noir (especially in vintages 2016, 2017) infected with Group X (diamond points) relative to Group I (star points) and Group VI (triangle points) virus infections. This reduced the overall percentage of GLRaV-3-infected vines positively identified by foliar symptoms. Figure S2: Visual identification of grapevine leafroll associated virus 3 (GLRaV-3) genotypes at the Marlborough site for the red-berry cultivars Merlot (left panel) and Pinot noir (right panel), in vintages 2015, 2016, and 2017. Generally, in all vintages, delayed symptom expression was observed for Merlot and Pinot noir (especially in vintages 2016, 2017) infected with Group X (diamond points) relative to Group I (star points) and Group VI (triangle points) virus infections. This reduced the overall percentage of GLRaV-3-infected vines positively identified by foliar symptoms, with the exception of vintage 2016, where the percentage of Group X-infected Merlot vines positively identified for disease based on foliar symptoms was greater than that for Group I in the same cultivar at the Julian day 53 (represents one extra vine identified). Figure S3: Foliar symptoms of the grapevine leafroll-associated virus 3 (GLRaV-3) Group X genotype generally appeared later than the Groups I and IV genotype symptoms from infected Pinot noir grapevines, as recorded in the Hawke’s Bay study site during vintages 2016 (left panel) and 2017 (right panel). For all genotypes, the expression of foliar symptoms moved from the laid cordon (base of the canopy) upwards, as the season progressed. Presented are the cumulative percentages of grapevines infected with genotypes Group I (star points), Group VI (triangle points), and Group X (diamond points) with observable leafroll symptoms in all basal canopy sectors (demarcated as Sections 1, 3, and 5), the first symptom appearance in the upper canopy sectors (demarcated as Sections 2, 4, and 6), and symptoms in all canopy sectors (demarcated Sections 1 to 6). Figure S4: Foliar symptoms of the grapevine leafroll-associated virus 3 (GLRaV-3) Group X genotype appeared later than the Groups I and IV genotype symptoms from infected Merlot grapevines, as recorded in the Auckland study site during vintages 2016 (left panel) and 2017 (right panel). For all genotypes, the expression of foliar symptoms moved from the laid cordon (base of the canopy) upwards, as the season progressed. Presented are the cumulative percentages of grapevines infected with genotypes Group I (star points), Group VI (triangle points), and Group X (diamond points) with observable leafroll symptoms in all basal canopy sectors (demarcated as Sections 1, 3, and 5), the first symptom appearance in the upper canopy sectors (demarcated as Sections 2, 4, and 6), and symptoms in all canopy sectors (demarcated Sections 1 to 6). Figure S5: Foliar symptoms of the grapevine leafroll-associated virus 3 (GLRaV-3) Group X genotype appeared later than the Groups I and IV genotype symptoms from infected Pinot noir grapevines, as recorded in the Auckland study site during vintage 2017. For all genotypes, the expression of foliar symptoms moved from the laid cordon (base of the canopy) upwards, as the season progressed. Presented are the cumulative percentages of grapevines infected with genotypes Group I (star points), Group VI (triangle points), and Group X (diamond points) with observable leafroll symptoms in all basal canopy sectors (demarcated as Sections 1, 3, and 5), the first symptom appearance in the upper canopy sectors (demarcated as Sections 2, 4, and 6), and symptoms in all canopy sectors (demarcated Sections 1 to 6). Figure S6: Visual identification of foliar symptoms from three canopy sections (V1, V2, V3) of Merlot (left panel) and Pinot noir (right panel) grapevines infected with either the GLRaV-3 Group I (star points), Group VI (triangle points), and Group X (diamond points) genotype, for the 2016 vintage visual assessments at the Hawke’s Bay site. No apparent difference in the observable foliar symptoms (either the first appearance of symptoms or full canopy coverage of symptoms) among the three vertical canopy sections was observed. Similar cumulative percentage of grapevines with observable symptoms from shoots closest to the trunk (V1) compared with shoots furthest away from the trunk (V3) throughout the growing season. Figure S7: Visual identification of foliar symptoms from three canopy sections (V1, V2, V3) of Merlot (left panel) and Pinot noir (right panel) grapevines infected with either the GLRaV-3 Group I (star points), Group VI (triangle points), and Group X (diamond points) genotype, for the 2017 vintage visual assessments at the Hawke’s Bay site. No apparent difference in the observable foliar symptoms (either the first appearance of symptoms or full canopy coverage of symptoms) among the three vertical canopy sections was observed. A similar cumulative percentage of grapevines with observable symptoms from shoots closest to the trunk (V1) compared with shoots furthest away from the trunk (V3) throughout the growing season. Figure S8: Visual symptom identification of grapevine leafroll associated virus 3 (GLRaV-3) from Merlot (left panel) and Pinot noir (right panel) grapevines infected with a single GLRaV-3 genotype (square points) or with two GLRaV-3 genotypes (circle points) at the Auckland site, for the 2015, 2016, 2017 vintages. Figure S9: Visual symptom identification of grapevine leafroll associated virus 3 (GLRaV-3) from Merlot (left panel) and Pinot noir (right panel) grapevines infected with only the GLRaV-3 Group I genotype (star points) and with Group I in a dual infection with Group VI (circle points filled with a “+”) or Group X (“X” points) genotypes at the Auckland site, for the 2016 and 2017 vintages. Figure S10: Visual symptom identification of grapevine leafroll associated virus 3 (GLRaV-3) from Merlot (left panel) and Pinot noir (right panel) grapevines infected with only the GLRaV-3 Group VI genotype (triangle points), and with Group VI in a dual infection with Group I (circle points filled with a “+”) or Group X (“” points) genotypes at the Auckland site, for the 2016 and 2017 vintages.
