# Supplementary material for: Grapevine Leafroll-Associated Virus 3 Genotype Influences Foliar Symptom Development in New Zealand Vineyards
Source: Viruses. 2022 Jun 21;14(7):1348. doi: 10.3390/v14071348 (PMC9316759; doi:10.3390/v14071348)
Supplement: Supplementary file 1 [file viruses-14-01348-s001.zip › viruses-1699557-supplementary.pdf]

Supplementary Materials for the manuscript titled "Grapevine leafroll-associated virus 3 genotype influences foliar symptom development in New Zealand vineyards"

Table S1: Detection of grapevine leafroll-associated virus 3 and other grapevine viruses and/or viroids by high throughput sequencing (HTS) in plant material from source plants used.

Plant ID	Expected GLRaV-3 genotype [1]	GLRaV-3 genotype(s) detected by HTS			Other viruses and/or viroids detected by HTS [2]	
		Group I	Group VI	Group X	GRSPaV	HSVd
10-24-3	Group I	+			+	+
19-5-5	Group VI		+		+	
19-14-5	Group X			+	+	
18-42-1	Group I + X	+		+	+	+
Pal 3	Group VI + X		+	+		
Pal 6	Group VI + X		+	+		

[1] Expected GLRaV-3 genotype based on conventional and real-time RT-PCR assays using GLRaV-3-specific Primers [26,27] screening.
[2] GRSPaV, Grapevine Rupestris stem pitting associated virus; HSVd, Hop stunt viroid.

Table S2: The success rate of the green-graft inoculations for each of the grapevine leafroll-associated virus 3 (GLRaV-3) infection types and grapevine cultivars.

GLRaV-3 infection type	Grapevine cultivar	Number of vines required	Number of vines green grafted	Number of successful grafted vines (%)[a]	Average success rate of green grafts (%)
Group I	Merlot	80	120	90 (75%)	79.0
	Pinot noir	80	120	93 (77.5%)	
	Pinot gris	80	120	96 (80%)	
	Sauvignon blanc	80	120	100 (83.3%)	
Group VI	Merlot	60	96	62 (64.6%)	76.0
	Pinot noir	60	96	64 (66.7%)	
	Pinot gris	60	96	86 (89.6%)	
	Sauvignon blanc	60	96	80 (83.3%)	
Group X	Merlot	60	96	75 (78.1%)	81.0
	Pinot noir	60	96	82 (85.4%)	
	Pinot gris	60	96	92 (95.8%)	
	Sauvignon blanc	60	96	62 (64.6%)	
Group I + X	Merlot	20	40	24 (60%)	84.4
	Pinot noir	20	40	40 (100%)	
	Pinot gris	20	40	39 (97.5%)	
	Sauvignon blanc	20	40	32 (80%)	
Group VI + X	Merlot	20	32	11 (34.4%)	30.5
	Pinot noir	20	32	10 (31.3%)	
	Pinot gris	20	32	4 (12.5%)	
	Sauvignon blanc	20	32	14 (43.8%)	

[a] The number within the brackets is the percentage success rate of the green-graft inoculation for GLRaV-3 infection type and cultivar.

Table S3: Total number of biological replicates for each dual grapevine leafroll-associated virus 3 genotype infection treatment and cultivar planted at the Auckland field trial site. Grapevines were planted at the Auckland location across two vintages, 2014 and 2015.

GLRaV-3 infection type/ Treatment	Merlot		Pinot noir		Pinot gris		Sauvignon blanc	
	2014	2015	2014	2015	2014	2015	2014	2015
Group I+VI	1	17	20	-	-	13	-	20
Group I+X	20	-	20	-	20	-	19	1
Group VI+X	11	9	9	11	4	13	13	6
Healthy	20	-	20	-	20	-	19	1
Overall total:	52	26	69	11	44	26	51	28

Table S4: Total number of biological replicates (Rep.), number of vines tested negative for virus after two consecutive years of laboratory testing (Neg.), and number of missing vines (Missing) for each treatment and cultivar in the Auckland, Hawke's Bay, and Marlborough grapevine leafroll-associated virus 3 (GLRaV-3) field trial sites.

GLRaV-3 infection type/ Treatment	Auckland			Hawke's Bay			Marlborough		
	Rep.	Neg.	Missing	Rep.	Neg.	Missing	Rep.	Neg.	Missing
				Merlot					
Group I	20	-	-	20	-	-	20	-	-
Group VI	20	-	-	19	-	1	20	-	-
Group X	20	-	-	19	-	1	19	1	-
Healthy	20	-	-	20	-	-	20	-	-
				Pinot noir					
Group I	20	-	-	20	-	-	18	1	1
Group VI	17	3	-	17	1	2	20	-	-
Group X	20	-	-	20	-	-	20	-	-
Healthy	20	-	-	20	-	-	20	-	-
				Pinot gris					
Group I	19	1	-	19	-	1	19	-	1
Group VI	19	-	1	20	-	-	17	-	3
Group X	16	4	-	16	4	-	19	-	1
Healthy	20	-	-	20	-	-	20	-	-
				Sauvignon blanc					
Group I	20	-	-	20	-	-	20	-	-
Group VI	20	-	-	19	-	1	19	-	1
Group X	20	-	-	20	-	-	20	-	-
Healthy	20	-	-	20	-	-	20	-	-
Overall total:	311	8	1	309	5	6	311	2	7

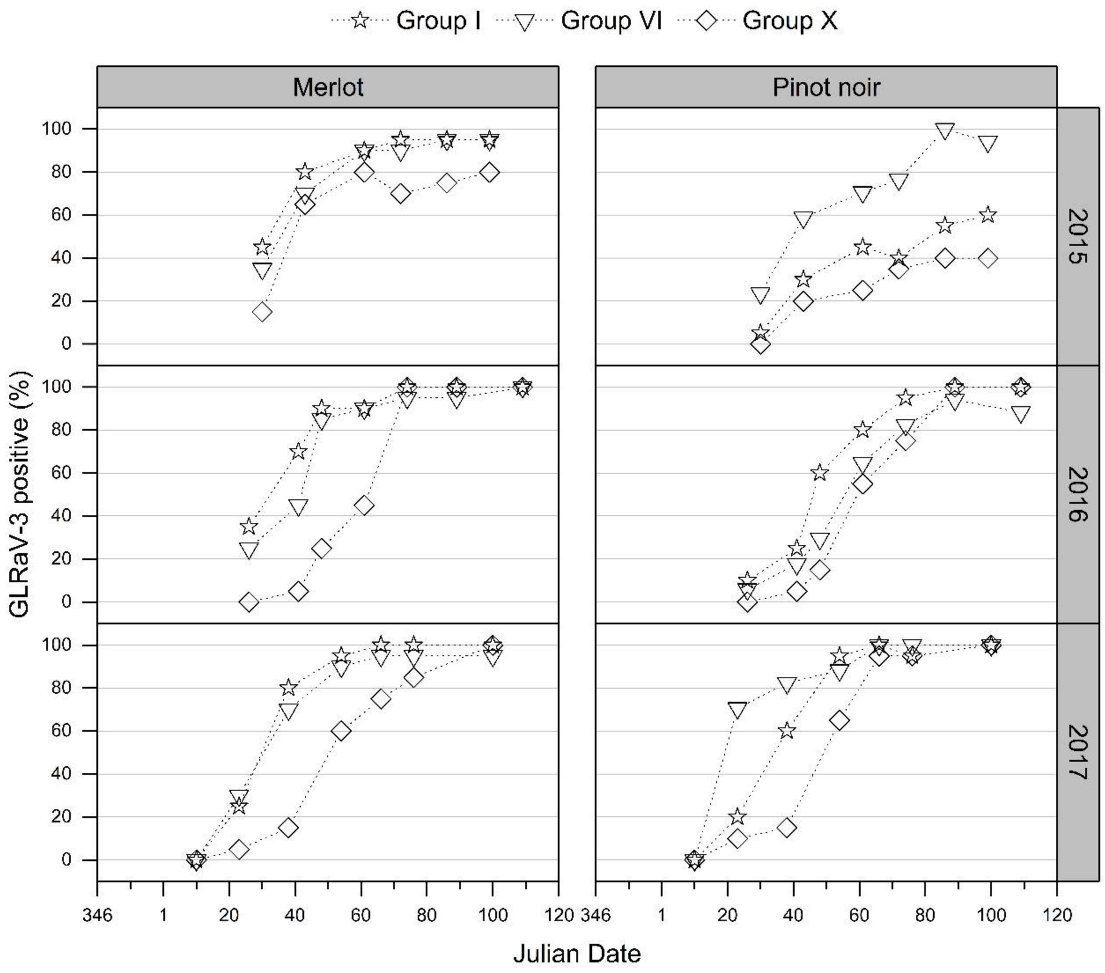

Figure S1: Visual identification of grapevine leafroll associated virus 3 (GLRaV-3) genotypes at the Auckland site for the red-berry cultivars Merlot (left panel) and Pinot noir (right panel), in vintages 2015, 2016, and 2017. Generally, in all vintages, delayed symptom expression was observed for Merlot and Pinot noir (especially in vintages 2016, 2017) infected with Group X (diamond points) relative to Group I (star points) and Group VI (triangle points) virus infections. This reduced the overall percentage of GLRaV-3-infected vines positively identified by foliar symptoms.

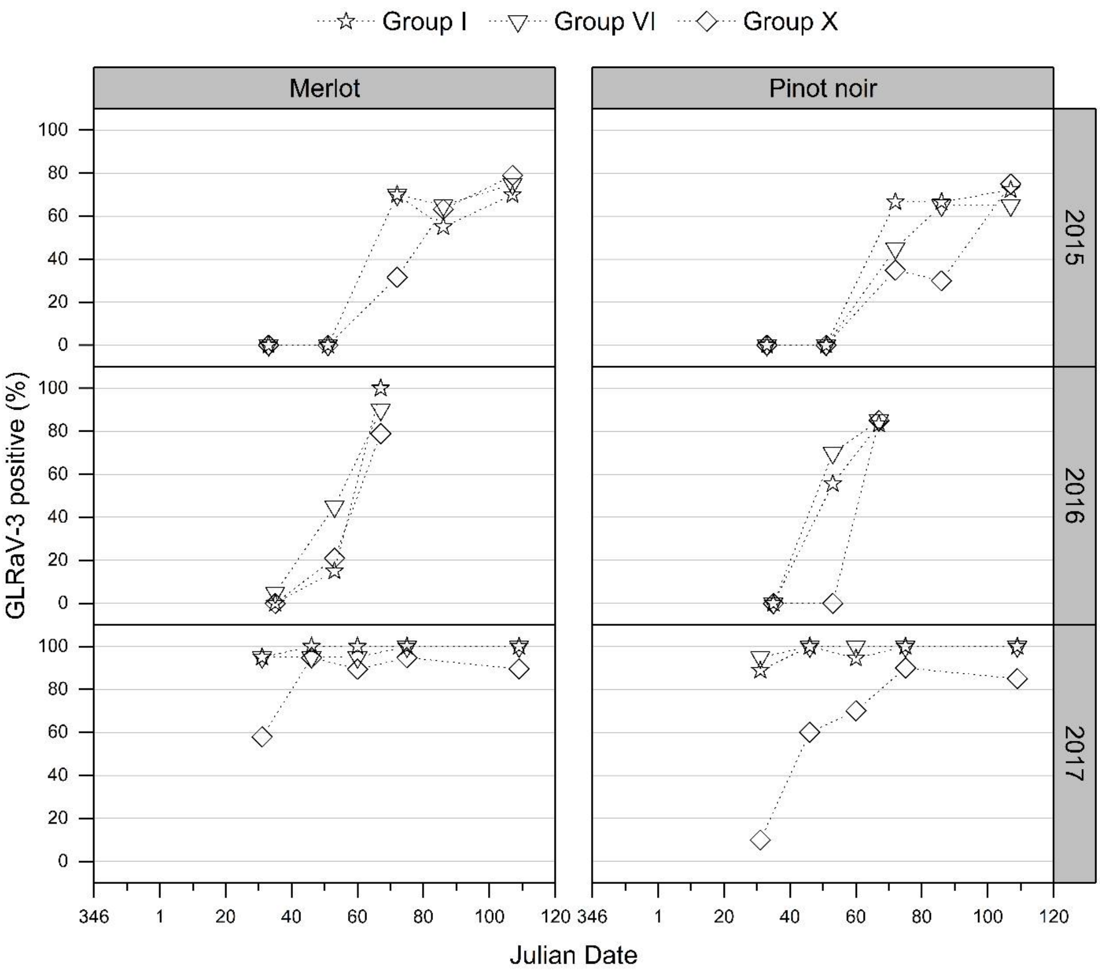

Figure S2: Visual identification of grapevine leafroll associated virus 3 (GLRaV-3) genotypes at the Marlborough site for the red-berry cultivars Merlot (left panel) and Pinot noir (right panel), in vintages 2015, 2016, and 2017. Generally, in all vintages, delayed symptom expression was observed for Merlot and Pinot noir (especially in vintages 2016, 2017) infected with Group X (diamond points) relative to Group I (star points) and Group VI (triangle points) virus infections. This reduced the overall percentage of GLRaV-3-infected vines positively identified by foliar symptoms, with the exception of vintage 2016, where the percentage of Group X-infected Merlot vines positively identified for disease based on foliar symptoms was greater than that for Group I in the same cultivar at the Julian day 53 (represents one extra vine identified).

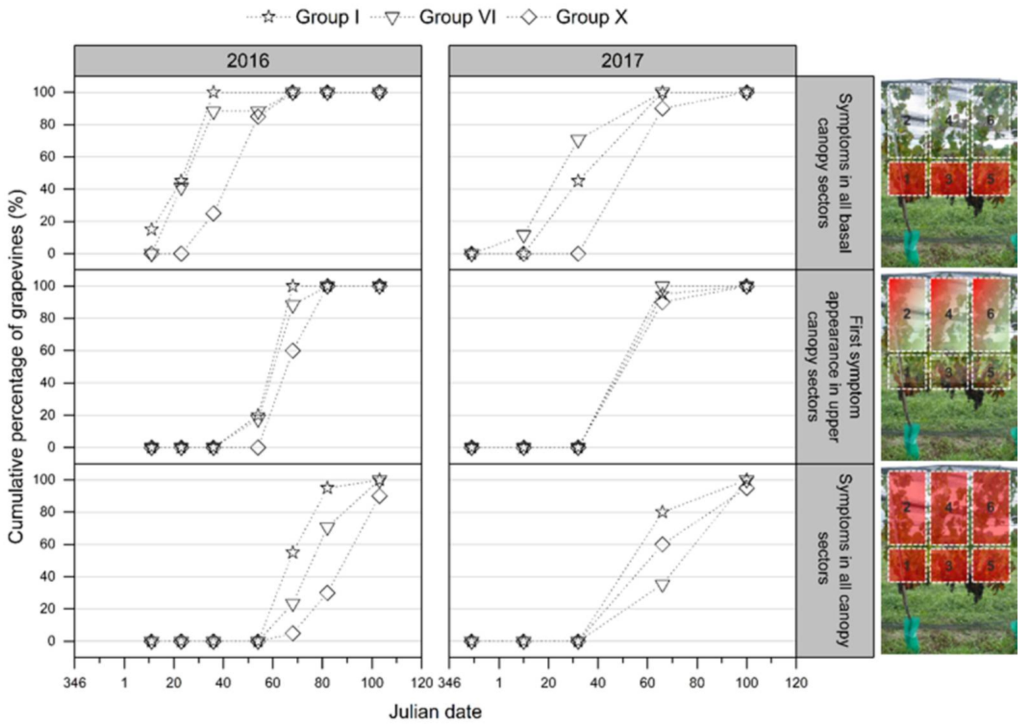

Figure S3: Foliar symptoms of the grapevine leafroll-associated virus 3 (GLRaV-3) Group X genotype generally appeared later than the Groups I and IV genotype symptoms from infected Pinot noir grapevines, as recorded in the Hawke's Bay study site during vintages 2016 (left panel) and 2017 (right panel). For all genotypes, the expression of foliar symptoms moved from the laid cordon (base of the canopy) upwards, as the season progressed. Presented are the cumulative percentages of grapevines infected with genotypes Group I (star points), Group VI (triangle points), and Group X (diamond points) with observable leafroll symptoms in all basal canopy sectors (demarcated as Sections 1, 3, and 5), the first symptom appearance in the upper canopy sectors (demarcated as Sections 2, 4, and 6), and symptoms in all canopy sectors (demarcated Sections 1 to 6).

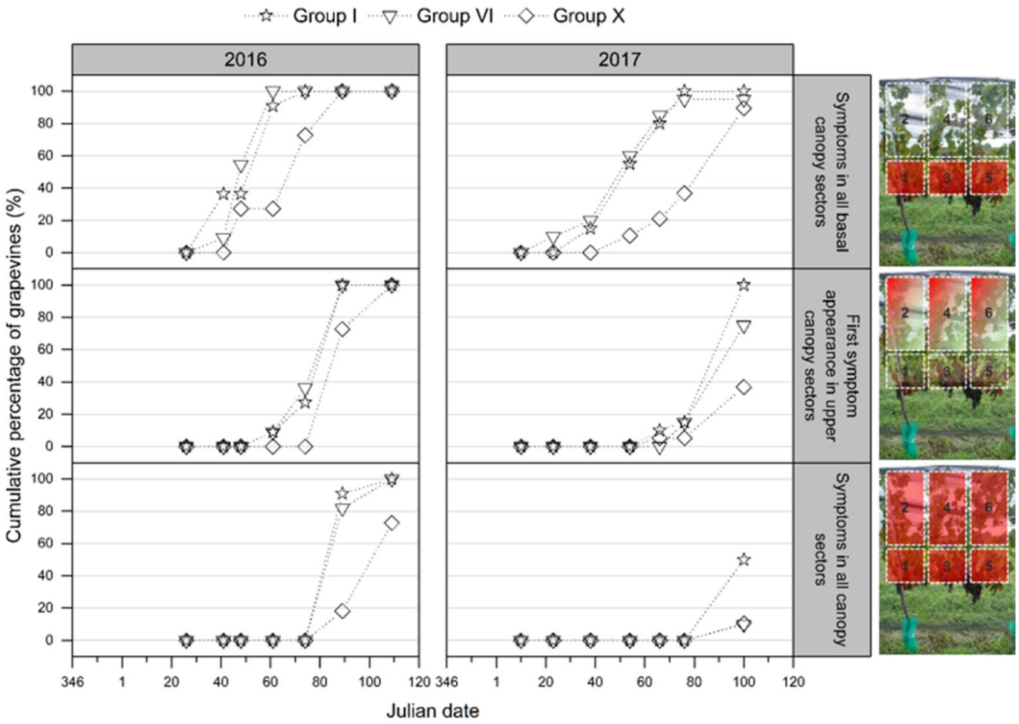

Figure S4: Foliar symptoms of the grapevine leafroll-associated virus 3 (GLRaV-3) Group X genotype appeared later than the Groups I and IV genotype symptoms from infected Merlot grapevines, as recorded in the Auckland study site during vintages 2016 (left panel) and 2017 (right panel). For all genotypes, the expression of foliar symptoms moved from the laid cordon (base of the canopy) upwards, as the season progressed. Presented are the cumulative percentages of grapevines infected with genotypes Group I (star points), Group VI (triangle points), and Group X (diamond points) with observable leafroll symptoms in all basal canopy sectors (demarcated as Sections 1, 3, and 5), the first symptom appearance in the upper canopy sectors (demarcated as Sections 2, 4, and 6), and symptoms in all canopy sectors (demarcated Sections 1 to 6).

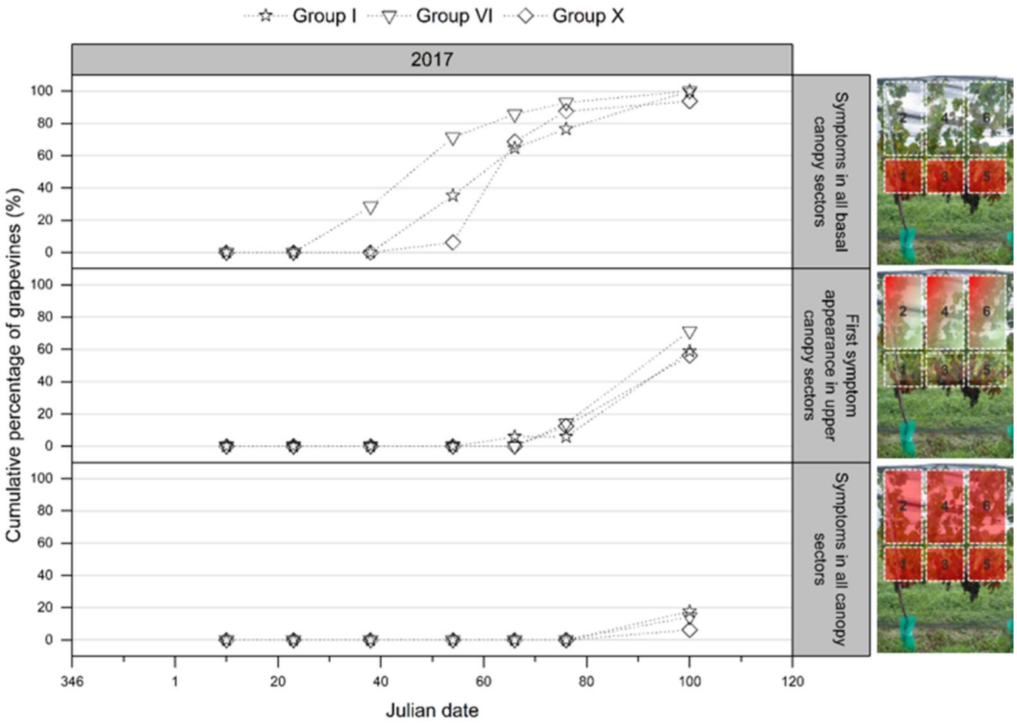

Figure S5: Foliar symptoms of the grapevine leafroll-associated virus 3 (GLRaV-3) Group X genotype appeared later than the Groups I and IV genotype symptoms from infected Pinot noir grapevines, as recorded in the Auckland study site during vintage 2017. For all genotypes, the expression of foliar symptoms moved from the laid cordon (base of the canopy) upwards, as the season progressed. Presented are the cumulative percentages of grapevines infected with genotypes Group I (star points), Group VI (triangle points), and Group X (diamond points) with observable leafroll symptoms in all basal canopy sectors (demarcated as Sections 1, 3, and 5), the first symptom appearance in the upper canopy sectors (demarcated as Sections 2, 4, and 6), and symptoms in all canopy sectors (demarcated Sections 1 to 6).

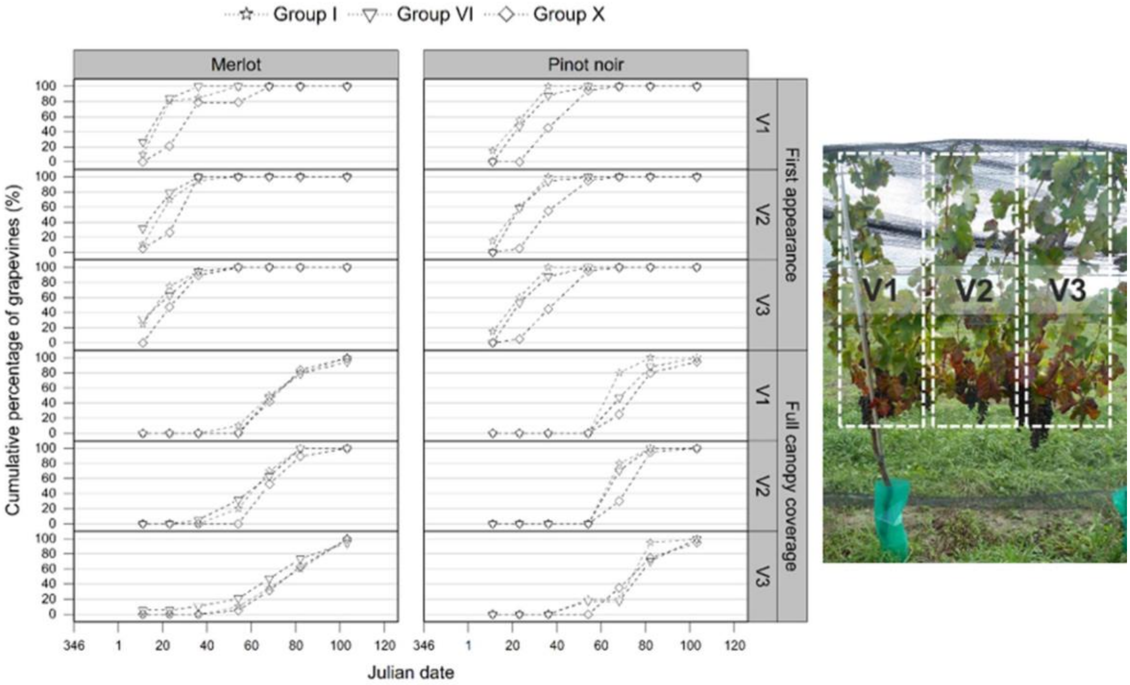

Figure S6: Visual identification of foliar symptoms from three canopy sections (V1, V2, V3) of Merlot (left panel) and Pinot noir (right panel) grapevines infected with either the GLRaV-3 Group I (star points), Group VI (triangle points), and Group X (diamond points) genotype, for the 2016 vintage visual assessments at the Hawke's Bay site. No apparent difference in the observable foliar symptoms (either the first appearance of symptoms or full canopy coverage of symptoms) among the three vertical canopy sections was observed. Similar cumulative percentage of grapevines with observable symptoms from shoots closest to the trunk (V1) compared with shoots furthest away from the trunk (V3) throughout the growing season.

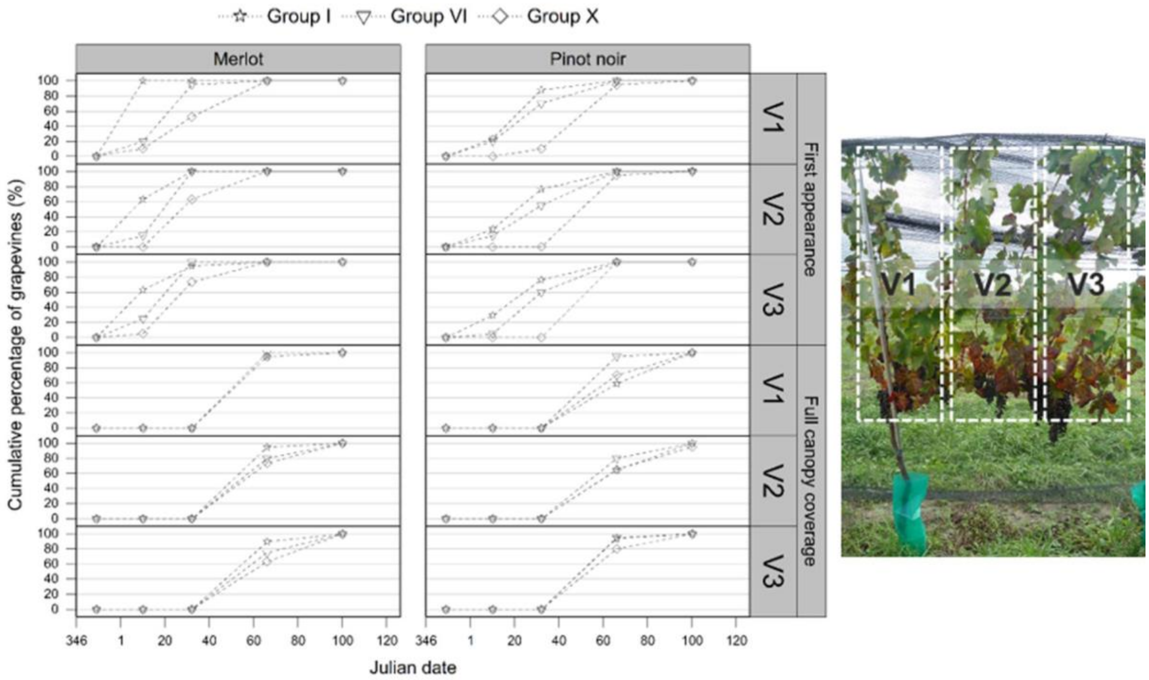

Figure S7. Visual identification of foliar symptoms from three canopy sections (V1, V2, V3) of Merlot (left panel) and Pinot noir (right panel) grapevines infected with either the GLRaV-3 Group I (star points), Group VI (triangle points), and Group X (diamond points) genotype, for the 2017 vintage visual assessments at the Hawke's Bay site. No apparent difference in the observable foliar symptoms (either the first appearance of symptoms or full canopy coverage of symptoms) among the three vertical canopy sections was observed. A similar cumulative percentage of grapevines with observable symptoms from shoots closest to the trunk (V1) compared with shoots furthest away from the trunk (V3) throughout the growing season.

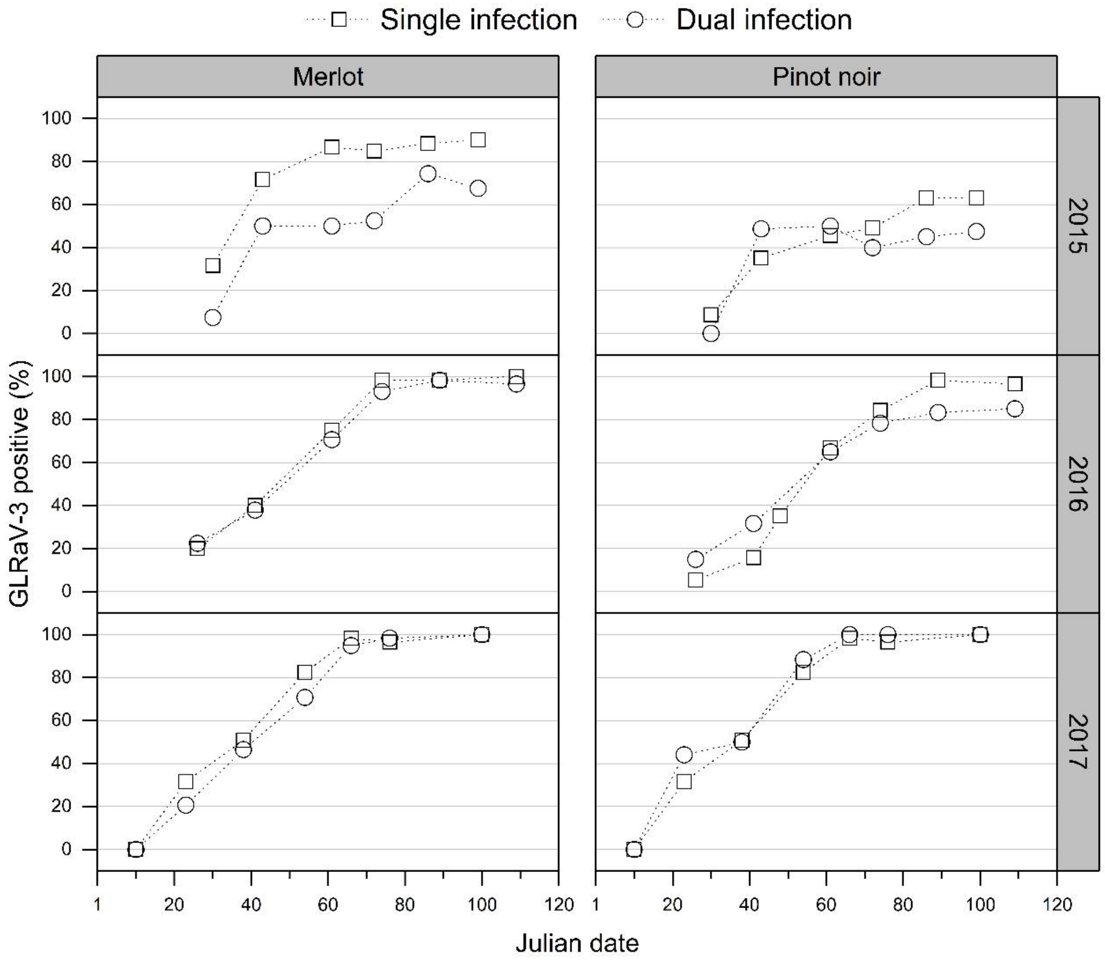

Figure S8: Visual symptom identification of grapevine leafroll associated virus 3 (GLRaV-3) from Merlot (left panel) and Pinot noir (right panel) grapevines infected with a single GLRaV-3 genotype (square points) or with two GLRaV-3 genotypes (circle points) at the Auckland site, for the 2015, 2016, 2017 vintages.

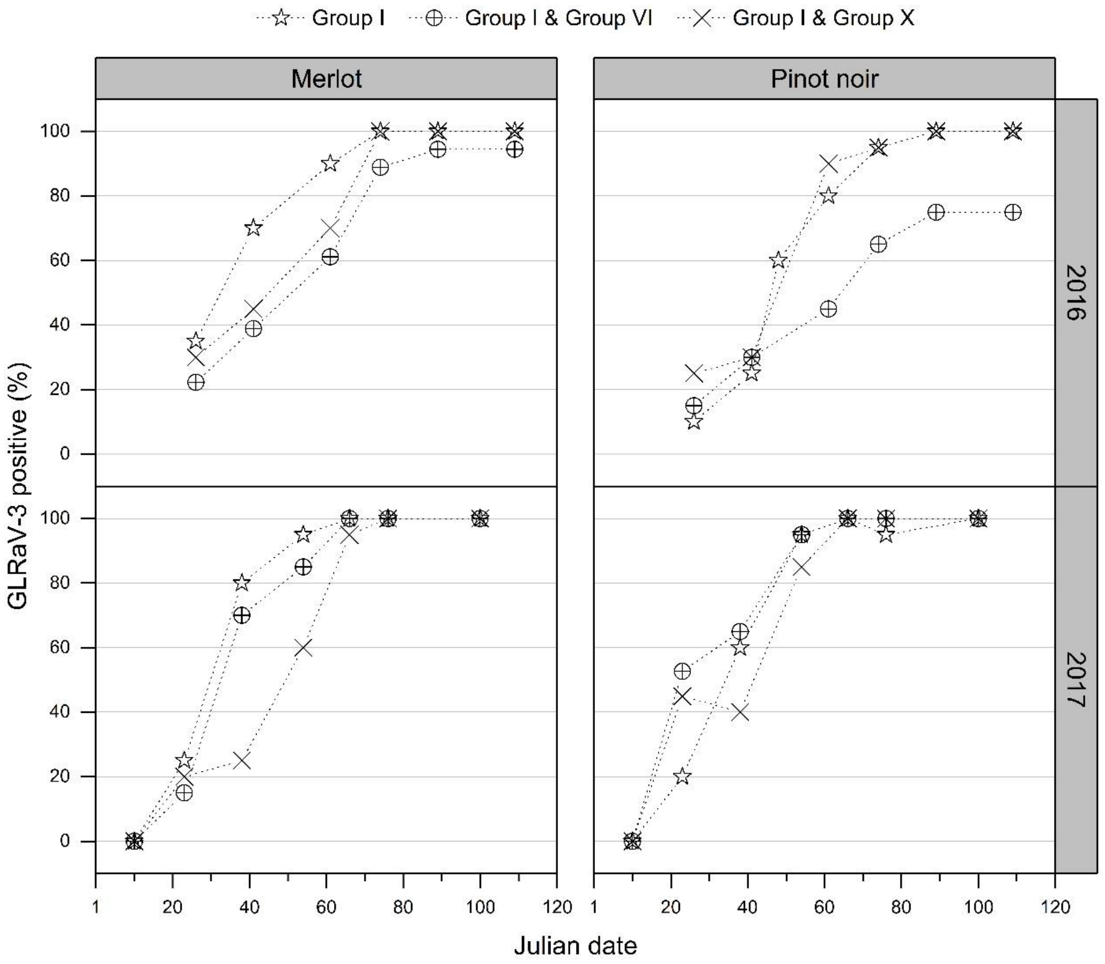

Figure S9: Visual symptom identification of grapevine leafroll associated virus 3 (GLRaV-3) from Merlot (left panel) and Pinot noir (right panel) grapevines infected with only the GLRaV-3 Group I genotype (star points) and with Group I in a dual infection with Group VI (circle points filled with a "+") or Group X ("X" points) genotypes at the Auckland site, for the 2016 and 2017 vintages.

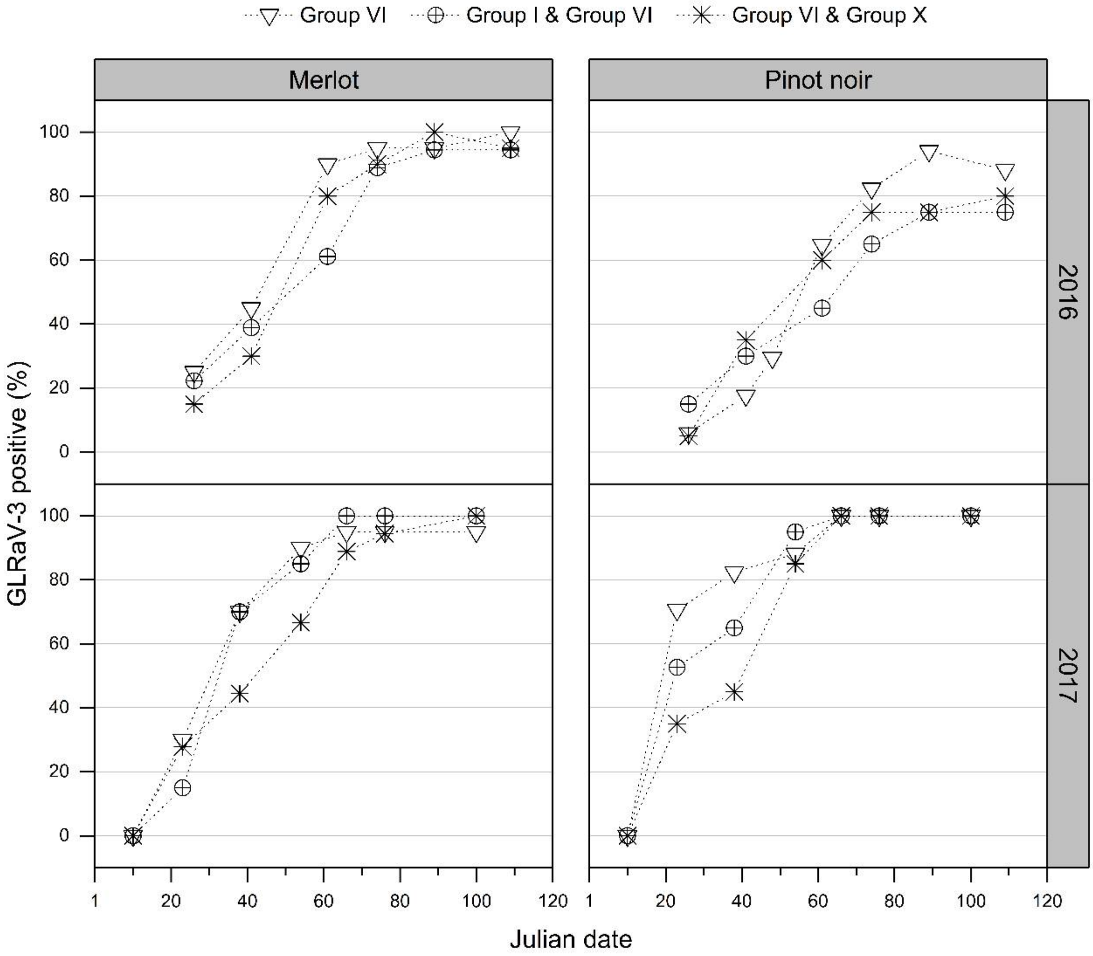

Figure S10: Visual symptom identification of grapevine leafroll associated virus 3 (GLRaV-3) from Merlot (left panel) and Pinot noir (right panel) grapevines infected with only the GLRaV-3 Group VI genotype (triangle points), and with Group VI in a dual infection with Group I (circle points filled with a "+") or Group X (" " points) genotypes at the Auckland site, for the 2016 and 2017 vintages.
